# Overexpression of the transcription factor ATF3 with a regulatory molecular signature associates with the pathogenic development of colorectal cancer

**DOI:** 10.18632/oncotarget.16638

**Published:** 2017-03-29

**Authors:** Feng Yan, Le Ying, Xiaofang Li, Bin Qiao, Qiaohong Meng, Liang Yu, Xiangliang Yuan, Shu-Ting Ren, David W Chan, Liyun Shi, Peihua Ni, Xuefeng Wang, Dakang Xu, Yiqun Hu

**Affiliations:** ^1^ Faculty of Medical Laboratory Science, Ruijin Hospital, Shanghai Jiaotong University School of Medicine, Shanghai, China; ^2^ Institute of Ageing Research, Hangzhou Normal University School of Medicine, Hangzhou, China; ^3^ Department of Tea Science, Zhejiang University, Hangzhou, China; ^4^ Department of Stomatology, The First Affiliated Hospital of Zhengzhou University, Zhengzhou, Henan, China; ^5^ Department of General Surgery, Shanghai Jiao Tong University Affiliated First People's Hospital, Shanghai, China; ^6^ Department of Pathology, School of Basic Medical Science, Xi’an Jiaotong University Health Science Center, Xi’an, China; ^7^ Department of Obstetrics and Gynaecology, LKS Faculty of Medicine, The University of Hong Kong, Hong Kong SAR, P.R. China; ^8^ Department of Microbiology and Immunology, Nanjing University of Chinese Medicine, Nanjing, China; ^9^ Department of Laboratory Medicine, Ruijin Hospital, Shanghai Jiaotong University School of Medicine, Shanghai, China; ^10^ Hudson Institute of Medical Research, Clayton, Victoria, Australia; ^11^ Department of Molecular and Translational Science, Monash University, Clayton, Victoria, Australia

**Keywords:** colorectal cancer, ATF3, genes signature, prognosis

## Abstract

The identification of novel biomarkers of cancer is important for improved diagnosis and prognosis. With an abundant amount of resources in the publicly available database, such as the Cancer Genome Atlas (TCGA) database, an integrative strategy is used to systematically characterize the aberrant patterns of colorectal cancer (CRC) based on RNA-Seq, chromatin immunoprecipitation sequencing (ChIP-Seq), tissue microarray (TMA), gene profiling and molecular signatures. The expression of the transcription factor ATF3 was elevated in human CRC specimens in a TMA by immunochemistry analysis compared to the adjacent normal tissues. In addition, ATF3 overexpression associated with a regulatory molecular signature, and its functions are related to the pathogenic development of CRC. Furthermore, putative ATF3 regulatory elements were identified within the promoters of *ATF3* target genes and were confirmed by ChIP-Seq. Critically, in higher ATF3 expression cell lines (HCT116 and RKO) with CRISPR/Cas9 mediated ATF3 knock out, we are able to show that ATF3 target genes such as *CEACAM1, DUSP14, HDC, HLF* and *ULBP2*, are required for invasion and proliferation, and they are robustly linked with poor prognosis in CRC. Our findings have important implications for CRC tumorigenesis and may be exploited for diagnostic and therapeutic purposes.

## INTRODUCTION

Colorectal cancer (CRC) is the third most common malignant neoplasm in western countries. The burden of CRC is increasing in the Asia-Pacific region [1]. Its prognosis has been elevated gradually due to developing procedures such as surgery, radiotherapy and chemotherapy. However, the survival rate of patients with CRC is still very low [2]. The identification of novel CRC biomarkers play an important role in making more selective treatment decisions.

Significant progress has been made in identifying novel molecular signatures to understand CRC subsets over the last few years. As a result, the Wnt signaling pathway, the RAS pathway, and functional loss of tumor suppressor genes (*p53* and *APC*) have been well understood [3]. To date, much effort has been made to identify biomarkers. However, it has achieved limited success because it focused on a single protein or gene mutation [4]. More gene signatures and signaling pathways of CRC are needed to develop a firmer understanding of CRC progression and to improve clinical prognosis and therapy.

Recently, high-throughput technologies have been developed, and a comprehensive method of integrating the information from different technological platforms including gene expression profiling and genomic sequencing analysis enables us to identify gene signatures and the related signaling pathways. Using patient tissue genomic, RNA expression data and clinical information, the Cancer Genome Atlas (TCGA) obtains an overview of cancer related profiles. It can also identify potential biomarkers for diagnosis diseases and prognosis. With the help of high-throughput sequencing technology, bioinformatic methods have been developed to identify differential expressed genes from RNA-Seq and correlate the regulation of regenerating genes by chromatin immunoprecipitation sequencing (ChIP-Seq). Additionally, the Kaplan–Meier overall survival (OS) rates are also integrated [5, 6]. The abundant evidence of genetic and expression alterations based on the huge number of patient samples, may help us identify the relationship between clinical phenotypes and genetic expressions more accurately.

The biological and clinical significance of an overexpressed transcription factor (Myc) as an oncogene and the loss of a transcription factor (*p53*) as a tumor suppressor is that they are dysregulated during the initiation, progression and metastasis of CRC [7]. However, *p53*- and Myc-independent regulation also exists in CRC signaling through other transcription factors, and thus, identifying the key transcription factors that regulate the molecular signature associated with CRC progression will provide useful predictive markers. The activating transcription factor (ATF) family includes many basic-region leucine zipper (bZIP) transcription factors [8]. The ATF/cyclic AMP response element-binding (*CREB*) family members include ATF1, ATF2, ATF3, ATF4, ATF5, ATF6, ATF7, B-ATF, CREB and CREM. The bZIP element is a common feature of these proteins. The basic region in this domain plays the role of specific DNA binding, with the leucine zipper region forming homodimers or heterodimers with other proteins that contain bZIP elements, such as the AP-1, C/EBP or Maf families of proteins [8]. Substantial evidence shows that ATF3 may play an important role as a host defense by balancing the proliferative and apoptotic signals that are related with the progression of cancer [9]. It can also play various roles in cancer development in different cell types and contexts [8, 10]. Importantly, controversial roles for ATF3 have been reported. In normal tissues, ATF3 promotes both apoptosis and cell proliferation [11], while in neoplasms it has been identified as either an oncogene or a tumor suppressor, depending on the context of tumors [10, 12]. Previous studies investigated a limited number of cell lines and small cohorts, which were not biologically relevant. Thus, the function of ATF3 in tumor progression, including metastasis, has not yet been fully characterized. Our recent study suggested that ATF3 might play a role in bladder cancer metastasis [13]. However, the role of ATF3 in either pro-metastasis or anti-metastasis signaling remains controversial. These discrepancies are due to the context-dependent function of ATF3. Therefore, the accurate role of ATF3 in initiation and progression of CRC is still unclear. However, the role of ATF3 in CRC is paradoxical, with both pro- and anti-tumorigenic roles suggested.

In the current study, we systematically integrated data from TCGA, GEO and the JASPAR database, using RNA-Seq, ChIP-Seq and TMA to identify gene signature. Target genes of ATF3 regulation which have an important impact on tumor phenotype and prognosis were validated by colorectal cell line experiments. Critically, we demonstrate that ATF3, with its target genes, is required for an invasion tumor cell phenotype and is robustly linked with poor prognosis in CRC.

## RESULTS

### Workflow for the identification of a potential prognostic biomarker in CRC

**Figure 1:**
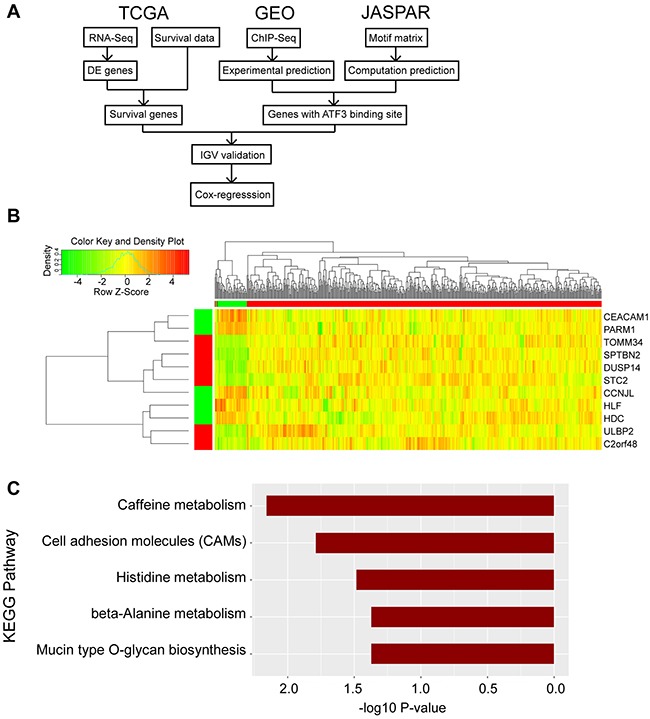
Identified the candidate genes for outcome of CRC **(A)** Flow-chart of the bioinformatic analysis in this research was shown. Data were collected from TCGA, GEO and JASPAR database. **(B)** Heatmap was generated in RStudio. The top right region represents the hierarchical clustering results, with red and green bars indicating tumor and adjacent normal samples, respectively. The left bottom region represents the 11 DEGs, with red and green bars indicating high and low expression in tumor samples compared to adjacent normal samples. **(C)** KEGG pathway analysis was conducted in RStudio and p value was shown.

We focused on tumor and adjacent normal tissue differences to develop a prognostic expression signature for CRC. Firstly, we used the TCGA RNA-Seq expression data to conduct a differential expression (DE) analysis and found differential expression genes (DEGs). Then, we combined the survival data from TCGA to screen the DEGs with a significant difference between the low and high expression subgroups. To validate the existence of ATF3 transcription factor binding sites in the promoter region, we used both the ChIP-Seq experimental data from GEO and the motif matrix from JASPAR. 47 genes were found to influence the survival rate with both a computational and experimental prediction of a binding site. We then validated the genes in Integrative Genomics Viewer (IGV) to find genes with the nearest binding site to transcription start site (TSS). Finally, we performed univariate and multivariate Cox regression to determine the significance level of the candidate genes (Figure 1A).

### Identification of candidate genes for CRC outcome

We investigated whether there was a significant difference in gene expressions between all the tumor (n = 478, male 252, female 226) and adjacent normal tissue (n = 41, male 20, female 21) samples using limma package in RStudio. In total, 2,086 genes were significantly differentially expressed between the tumor and adjacent normal tissue in both males and females; 527 (25.26%) genes were upregulated in both male and female tumor tissues, while 1,559 (74.74%) genes were downregulated. For most genes within this signature, there was a decreasing pattern of expression from the adjacent normal tissue to the tumor tissue.

Among all the DEGs, we conducted a Kaplan–Meier survival analysis that select target genes. We used the Benjamini & Hochberg method [14] to conduct multiple comparison corrections, but it was too conservative (data not shown). Since our downstream analysis and experiments can further validate the false positives, we used the unadjusted p values, and 148 genes had significantly different survival rates between the high- and low-expression subgroups. 44 (29.73%) genes were upregulated in the tumor, while 104 (70.27%) genes were downregulated.

### ATF3 presented a binding motif in a subset of candidate genes

**Figure 2:**
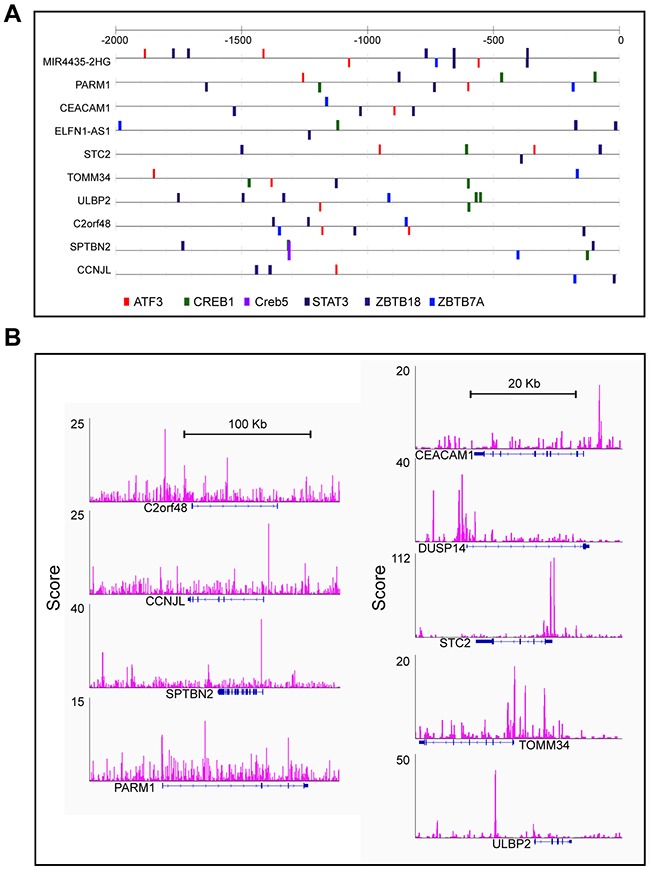
ATF3 presented binding motif in subset candidate genes **(A)** Computational predicting binding sites of ATF3 and other transcription factors were shown in the up-stream of these genes. **(B)** Experimental results of ATF3 ChIP-seq and peaks for ATF3 binding sites were show in Integrative Genomics Viewer (IGV).

A total of 2,086 DEGs were input as targets and another 1,068 least DEGs that had a log-fold-change (log2FC) <0.05 were used as background to eliminate the noise due to biological variance. In total, 1,755 genes had at least 1 predicted ATF3 binding site in our computational prediction. ATF3 was one of the top overrepresented transcription factors, and those signature genes had ATF3 binding sites in their upstream sequences. As a cancer prognostic marker, transcription factor STAT3 also presented binding sites (Figure 2A).

Meanwhile, the data from ATF3 ChIP-Seq were downloaded from the GEO database (GSM1917774_1152) for the control group to identify the experimental ATF3 binding site. The ATF3-WT HCT116 CRC cell line was used for the ChIP analysis, and 14,438 genes had a peak within a certain range from the TSS. We compared these genes (14438 genes) with the outcome genes from the computational prediction (1755 genes), and 854 genes overlapped with different expressions. Then, we compared the 854 genes with the 148 genes with a different survival rate; 47 genes overlapped. Information on these 47 genes, includes the logFC, p value and adjusted p value for male and female groups, p values for survival rates and distance to TSS was listed in Supplementary Table 1. Furthermore, up to 11 genes were identified with a regulated signature after further filtering. Only 9 genes (*CEACAM1, PARM1, TOMM34, SPTBN2, DUSP14, STC2, CCNJL, ULBP2*, and *C2orf48*) had a peak within 10 kbp from TSS, in both the computational prediction group and the experimental group (Figure 2B). In addition, 2 genes (*HLF* and *HDC*), which were reported to interact with ATF3, had peaks but were either far from the TSS or in the intron. We also identified the peaks for the 11 selected ATF3 target genes in another experiment from the ENCODE database (GSM1010757), and observed that the ChIP-Seq of ATF3 in HCT-116 cell line with 2 replicates (data not shown). Those 11 genes were more likely targeted by ATF3. The expression pattern of the 11 genes is shown in heatmap (Figure 1B). A KEGG pathway analysis was conducted for the 47 genes and the most significant pathways were related to cell adhesion molecules and histidine metabolism (Figure 1C).

By integrated data analysis from TCGA database between the tumor and adjacent normal tissues, we subsequently identified candidate genes for CRC outcome. The results were also confirmed by the ChIP-Seq from the public GEO database [15]. These 11 specific ATF3 target genes had very noticeable differences between cancer and adjacent normal tissues. There are also some genes that are expressed differently between various clinical stages (such as *STC2* and *ULBP2*), but most of the expressions between the stages (Supplementary Figure 1) only have a minimal change. ATF3 and the expressions of its target genes will be studied with further validation in a larger number of tissues and cell lines to assess their expressions and function.

### ATF3 regulated target genes in human CRC cells

**Figure 3:**
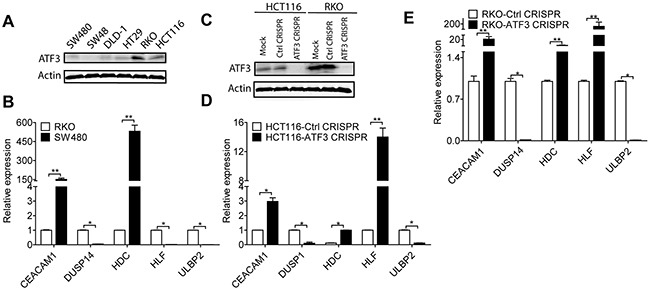
ATF3 regulated target genes **(A)** Representative images of ATF3 expression in CRC cell lines: RKO and HCT116 expressed higher ATF3 protein level by Western blot analysis. **(B)** *CEACAM1, DUSP14, HDC, HLF* and *ULBP2* mRNAexpressions were detected by qRT-PCR analysis. **(C)** RKO and HCT116 cells were transfected with ATF3 sgRNA, without doxycycline hyclate treatment as sgRNA CRISPR control, and with doxycycline hyclate treatment as CRISPR ATF3, which was confirmed by Western blot. **(D, E)** Silencing ATF3 caused changes in its target genes expressions by qRT-PCR analysis in HCT116 and RKO. Data represent mean ± SEM, (n=3). Paired t test in GraphPad Prism was used for data analysis. * represents p < 0.05. ** represents p < 0.01.

To further validate ATF3 and the expressions of its target genes, ATF3 protein expressions were studied in 6 human CRC cell lines. Western blot results revealed constitutively higher ATF3 expression in two cell lines (HCT116 and RKO), whereas there was no or lower expression in the colorectal cells (SW480, SW48, DLD-1 and HT29) (Figure 3A). RKO and SW480 cells were used to compare the mRNA expression of ATF3-related genes, and the results suggested that RKO, which had higher ATF3 protein expression, exhibited higher mRNA expression of *DUSP14, HLF*, and *ULBP2*. Moreover, a negative correlation between ATF3 expression and the expression of *CEACAM1* and *HDC* was observed comparing RKO and SW480 cells (Figure 3B). Those two genes are functionally important in CRC progression. *CEACAM1* is a metastasis-associated protein and histidine decarboxylase (*HDC*) is involved in the growth of colon tumors. Meanwhile, deleting ATF3 by CRISPR/Cas9 genome editing in the ATF3 high-expressing cell lines (HCT116 and RKO) altered expression of ATF3 and its target genes, such as *CEACAM1, DUSP14, HDC, HLF* and *ULBP2* (Figure 3C, 3D, 3E) may indicate the function in invasion and proliferation for further experiments.

Since the SW480 cell line had a lower ATF3 protein expression than RKO, some relative genes may be expressed differently. In SW480, there originally is a lower expression of *HLF* mRNA and may not be reflected by a low ATF3 status, although it was elevated in both ATF3 CRISPR cells in RKO and HCT116. Gene expressions of the other 6 genes in our experiments were less dependent on ATF3 expression, which was shown in the Supplementary Figure 2. The ATF3 mRNA expression of these cell lines was also detected by qRT-PCR and consistently aligns with the results of ATF3 protein expression (Supplementary Figure 2).

### ATF3 regulated target genes as an oncogenic function

**Figure 4:**
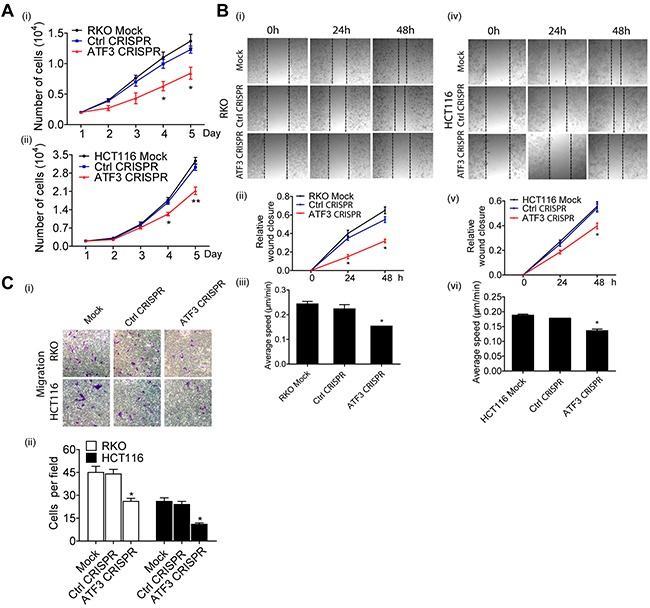
Functional characterization of ATF3 expression related to migration in CRC **(A)** Cell proliferation was determined by PrestoBlue Kit. **(A-i)** Significant difference was found in the numbers of RKO Mock cells, Control CRISPR cells and ATF3 CRISPR cells on day 4 and day 5. **(A-ii)** Significant difference also was found in the numbers of HCT116 Mock cells, Control CRISPR cells and ATF3 CRISPR cells on day 4 and day 5. **(B)** For wound healing assay, transfected cells were wounded by scratching and monitored over 24 hours to determine the rate of wound closure. Representative images of triplicate experiments are shown **(B-i and B-iv)** (10x). Cell migration of RKO and HCT116 cell lines were assessed by measuring relative wound closure **(B-ii and B-v)** and average speed **(B-iii and B-vi)**. **(C)** The metastatic properties of RKO and HCT116 cell lines and their transfected cells. Three independent experiments with three fields for each were performed and representative fields were shown (10x). Data represent mean ± SEM, (n=3). Paired t test in GraphPad Prism was used for data analysis. * represents p < 0.05. ** represents p < 0.01.

Cell proliferation, migration and invasion are the most important cell functions in the initial progression of cancer that relates with metastasis. To determine the contribution of ATF3 in CRC cell growth and motility, ATF3 CRISPR cells were assessed by wound healing assay and Transwell assay. There was no significant difference in cell proliferation in the first 24 h while ATF3 CRISPR cells grew more slowly in day 4 and 5 (Figure 4A). The migration of ATF3 CRISPR cells into the wounded area was significantly reduced compared to the control group (Figure 4B). RKO and HCT116 ATF3 CRISPR cells exhibited a significantly lower average velocity. These data demonstrated that ATF3 plays a key regulatory role in cell migration. The ATF3 CRISPR cells also exhibited reduced invasion ability by 50-60% compared to the control group (Figure 4C). These results showed that ATF3 regulates cell proliferation *in vitro*. Our RKO and HCT116 results were consistent with the other two independent studies, where ATF3 appeared to promote tumor growth and migration in HT29 cell line by either ATF3 antisense oligonucleotide or adenovirus-mediated overexpression of ATF3[16, 17].

### The expression of ATF3 and its target genes correlated with major clinical parameters of CRC

**Figure 5:**
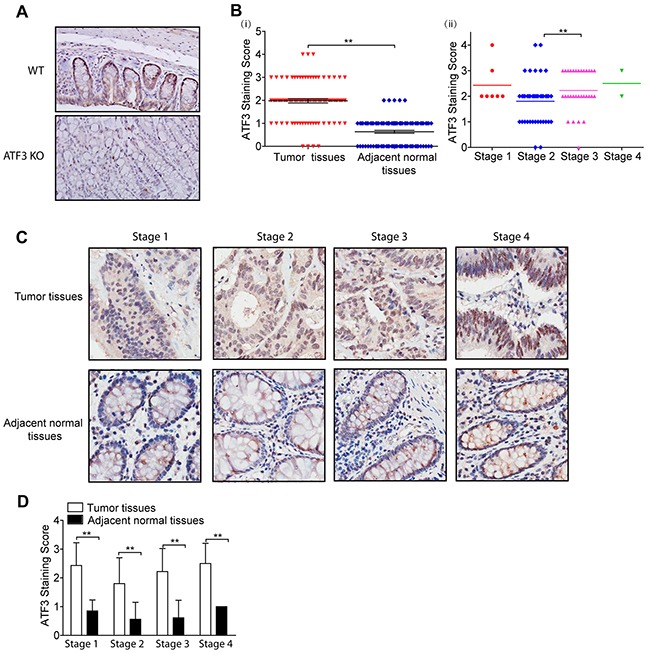
ATF3 is upregulated and positively correlated with stage in CRC **(A)** ATF3 immunohistochemical staining images of colon tissues from Wild type (WT) and ATF3 null (KO) mice (20x). **(A-i)** ATF3 staining scores of CRC tumor tissues and adjacent normal tissues from TMA. **(A-ii)** ATF3 staining scores of CRC tumor tissues in different clinical stages. Paired t test in GraphPad Prism was used for analysis in **(B-i)** and unpaired t test with Welch's correction in GraphPad Prism was used for analysis in **(B-ii)**. ** represents p < 0.01. **(C)** ATF3 staining images of different clinical stages (40x). **(D)** ATF3 staining scores of tumor tissues and adjacent normal tissues in different clinical stages. Paired t test in GraphPad Prism was used to compare the differences between the ATF3 staining scores of tumor tissues and adjacent normal tissues. ** represents p < 0.01.

To further study the potential clinical relevance of ATF3 to cancer progression, a TMA from CRC patients was developed to compare ATF3 expressions in CRC tissues and adjacent normal tissues. To confirm the specificity of the ATF3 antibody, we tested the ATF3 antibody in mice colon tissues. ATF3 staining was present more intensely in the nucleus than in the cytoplasm and was undetected in ATF3 null mice, which suggested that the antibody specifically recognizes ATF3 in the tissues (Figure 5A). The whole slide image of the TMA was attached as Supplementary Figure 3, which makes the analysis of the IHC results more comparable since they were scanned simultaneously and automatically. In 81 cases, higher ATF3 expression was observed in the CRC tissues compared to the adjacent normal tissues (28.3% vs. 0%, χ^2^=26.806, p<0.001; Supplementary Table 2 and Figure 5B, i). The ATF3 staining scores in clinical stages 3 were significantly higher than that of stage 2 (Figure 5B, ii). However, the ATF3 expression of stage 1 and stage 4 both seemed higher than that of stage 2 but there was no significant difference according to the results of unpaired t test, which may be due to the small sample sizes in stage 1 and 4. In different clinical stages, ATF3 staining score was much higher in the tumor tissues than in the adjacent normal tissues using paired t tests (Figure 5C, 5D), which suggested that ATF3 expression increased significantly in the tumor tissue compared with the adjacent normal tissues. Although the average ATF3 staining score in stage 3 and 4 were barely higher than that in stage 2 of Figure 5D, scores in clinical stage 3 was significantly higher than that of stage 2 based on unpaired t testing with Welch's correction analysis.

The association between ATF3 expression from the immunohistochemistry staining and the clinicopathological characteristics in CRC patients is summarized in Table 1. A Pearson χ^2^ analysis showed a significant correlation between ATF3 expression and the clinical stage (p=0.020), N stage (p=0.020), and patient survival status (p=0.039). However, no significant correlation was found between ATF3 expression and other clinical features, such as age, gender, tumor size, etc.

**Table 1:** Association between ATF3 expression and clinicopathological characteristics in colon cancer patients

Characteristic	n	ATF3- (%)	ATF3+ (%)	Pearson χ^2^	p value
Total	81				
Gender				0.100	0.752
Male	41	30 (73.17)	11 (26.83)		
Female	40	28 (70.00)	12 (30.00)		
Age				2.674	0.096
<70	38	31 (81.58)	7 (18.42)		
≥70	43	28 (65.12)	15 (34.88)		
Clinical stage				5.391	0.020*
I+II	48	39 (81.25)	9 (18.75)		
III+IIII	33	19 (57.58)	14 (42.42)		
T stage				1.283	0.257
T1+T2	9	5 (55.56)	4 (44.44)		
T3+T4	72	53 (73.61)	19 (26.39)		
N stage				5.391	0.020*
N0	48	39 (81.25)	9 (18.75)		
N1+N2	33	19 (57.58)	14 (42.42)		
M stage				0.471	0.493
M0	79	57 (72.15)	22 (27.85)		
M1	2	1 (50.00)	1 (50.00)		
Patient status				4.234	0.039*
live	32	27 (84.38)	5 (15.62)		
death	49	31 (63.27)	18 (36.73)		
Tumor size				0.031	0.86
<37.5	40	29 (72.50)	11 (27.50)		
≥37.5	41	29 (70.73)	12 (29.27)		
Tumor site				0.995	0.803
Right hemicolon	27	19 (70.37)	8 (29.63)		
Transverse colon	13	8 (61.54)	5 (38.46)		
Left hemicolon	12	9 (75.00)	3 (25.00)		
Sigmoid colon	29	22 (75.86)	7 (24.14)		

* p < 0.05, ** p < 0.01.

To test whether ATF3 expression might be a prognostic predictor in CRC, we used a Cox regression analysis to examine ATF3 expression and other clinicopathological characteristics with OS in 81 CRC patients. A univariate analysis indicated that ATF3 expression (HR 0.454; 95% CI 0.252-0.817; p=0.008), clinical stage (HR 2.354; 95% CI 1.336-4.148; p=0.003), N stage (HR 2.230; 95% CI 1.539-3.497; p<0.001), M stage (HR 9.851; 95% CI 2.233-43.466; p=0.003), and tumor size (HR 0.520; 95% CI 0.295-0.919; p=0.024) were significantly correlated with OS (Table 2). The number of patients in the M stage group was very small, and thus, a further multivariate analysis did not include the M stage analysis. The results of the multivariate analysis revealed that ATF3 expression, clinical stage and N stage might be independent prognostic indicators of OS in CRC patients. These findings indicate the possibility of using high expression levels of ATF3 as a predictor of prognosis and survival.

**Table 2:** Univariate and multivariate analysis of prognostic factors for overall survival in colon cancer patients

	Univariate analysis			Multivariate analysis		
	HR	95% CI	p value	HR	95% CI	p value
ATF3 expression						
High versus low	0.454	0.252-0.817	0.008**	0.462	0.224-0.875	0.018*
Gender						
Male versus female	0.723	0.410-1.273	0.261			
Age (years)						
<70 versus ≥70	1.740	0.973-3.115	0.062			
Clinical stage						
I + II versus III + IIII	2.354	1.336-4.148	0.003**	0.191	0.080-0.458	<0.001**
T						
T1+T2 versus T3+T4	1.266	0.501-3.196	0.618			
N						
N0 versus N1+N2	2.230	1.539-3.497	<0.001**	0.308	0.127-0.746	0.009**
M						
M0 versus M1	9.851	2.233-43.466	0.003**			
Tumor size						
<37.5 versus ≥ 37.5	0.520	0.295-0.919	0.024*	0.476	0.261-0.869	0.016

* p < 0.05, ** p < 0.01.

HR hazard ratio; 95% CI 95% confidence interval.

### Evaluation of ATF3 and its target genes as a prognostic marker of CRC

**Figure 6:**
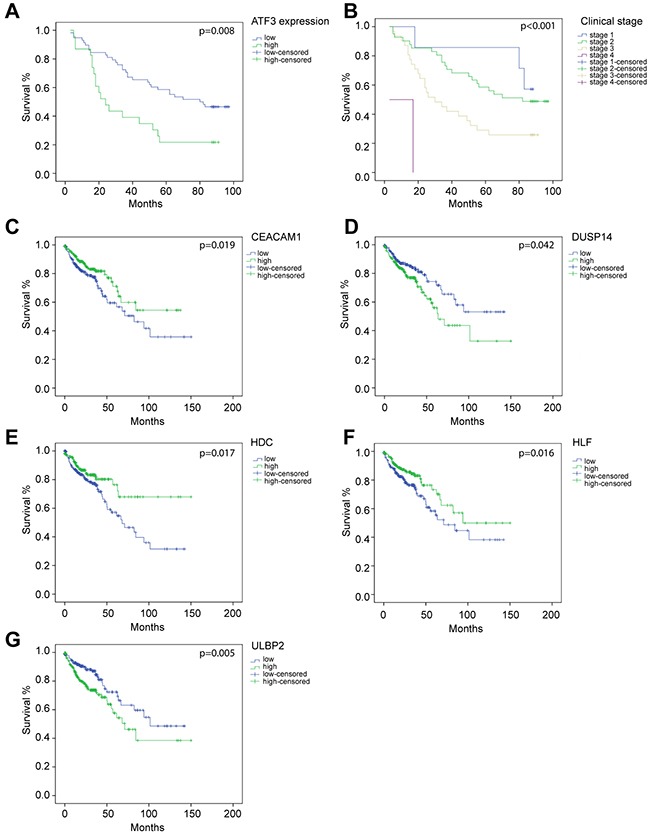
molecular signature associates genes with their survival analysis Overall survival curves by Kaplan–Meier method assessed for **(A)** ATF3 expression based on immunohistochemistry staining (p=0.007), **(B)** different clinical stages (p<0.001), **(C)** *CEACAM1* mRNA expression (p=0.037), **(D)** *DUSP14* mRNA expression (p=0.022), **(E)** *HDC* mRNA expression (p=0.015), **(F)** *HLF* mRNA expression (p=0.026), **(G)** *ULBP2* mRNA expression (p=0.004). (A-B) were based on the results of immunohistochemistry. (C-G) were based on data from TCGA database.

ATF3 and its target genes were associated with poor prognosis. Survival data based on the immunohistochemistry results showed that high ATF3 expression correlated with lower survival rates (p=0.008) (Figure 6A), and all the patients of different clinical stages had significantly different survival rates (p<0.001) (Figure 6B).

11 ATF3 candidate genes were analyzed by a Cox regression analysis, which indicated that all the ATF3 candidate genes were significantly associated with OS (Table 3) by a univariate analysis. Furthermore, 5 target genes of ATF3, including *CEACAM1, DUSP14, HDC, HLF*, and *ULBP2*, were highly related to OS in the clinical data (Figure 6C, 6D, 6E, 6F, 6G). Low levels of *CEACAM1, HDC*, and *HLF* expression were associated with poor prognosis (*CEACAM1* p=0.019; *HDC* p=0.017; *HLF* p=0.016). However, high levels of *DUSP14* and *ULBP2* were also related to a low survival rate of the patients (*DUSP14* p=0.042; *ULBP2* p=0.005). The Kaplan–Meier curve for overall survival rates assessed for the ATF3 mRNA expressions are shown in Supplementary Figure 4. There was no significant relevance between ATF3 mRNA expressions from the database and OS rate. A further multivariate analysis suggested that *ULBP2* (p=0.002), *HDC* (p=0.024) and *HLF* (p=0.022) were significant favorable prognostic genes of OS compared to the other ATF3 candidate genes. We also performed a Cox regression analysis taking stage as covariate, where most of the results were consistent with the previous study (data not shown).

**Table 3:** Univariate and multivariate analysis of target genes for overall survival in colon cancer patients

	Univariate analysis			Multivariate analysis		
	HR	95% CI	p value	HR	95% CI	p value
SPTBN2						
Low versus high	0.671	0.453-0.994	0.045*	0.817	0.543-1.229	0.331
DUSP14						
Low versus high	0.667	0.450-0.989	0.042*	0.743	0.496-1.114	0.151
CCNJL						
Low versus high	1.488	1.005-2.204	0.046*	1.447	0.967-2.165	0.072
CEACAM1						
Low versus high	1.597	1.076-2.37	0.019*	1.506	0.997-2.276	0.052
STC2						
Low versus high	0.675	0.456-0.998	0.047*	0.706	0.472-1.056	0.090
ULBP2						
Low versus high	0.569	0.383-0.845	0.005**	0.515	0.338-0.784	0.002**
TOMM34						
Low versus high	0.642	0.432-0.954	0.027*	0.668	0.432-1.033	0.069
C20rf48						
Low versus high	0.63	0.425-0.934	0.020*	0.734	0.486-1.108	0.141
PARM1						
Low versus high	1.539	1.037-2.284	0.031*	1.295	0.854-1.965	0.224
HDC						
Low versus high	0.615	1.088-2.427	0.017*	1.609	1.064-2.433	0.024*
HLF						
Low versus high	0.615	1.092-2.424	0.016*	1.602	1.070-2.398	0.022*

* p < 0.05, ** p < 0.01.

HR hazard ratio; 95% CI 95% confidence interval.

## DISCUSSION

CRC is associated with a high recurrence and a high mortality rate due to lack of knowledge about the gene signatures related to its pathogenic development. Thus, identifying key transcription factors that regulate the molecular signature associated with CRC progression will be useful. Here, an integrative strategy was used to comprehensively analyze the variable patterns of CRC based on RNA-Seq, ChIP-Seq, TMA, gene profiling and molecular signature. We studied the clinical and functional significance of ATF3 expression in CRC. An analysis of TMA showed that an increased ATF3 expression was related with CRC progression and a reduced survival rate in CRC patients. Correspondingly, depletion of ATF3 in high metastatic potential CRC cell lines decreased cell migration and proliferation. In agreement with their increased motility, metastatic CRC cells are associated with controlled inhibition of the epithelial-mesenchymal transition (EMT). Moreover, the expression levels of ATF3 in CRC correlated with the expression of its target genes, such as *CEACAM1, DUSP14, HDC, HLF* and *ULBP2*, which are required for tumor cell invasion and proliferation and are robustly linked to poor prognosis in CRC.

ChIP-Seq and transcriptome analysis are progressively becoming available for molecular profiling in various cancers. The current challenge is to combine these various molecular biology information to obtain a more comprehensive and informative view of the key biological processes in cancer progression [18]. Our study aims to systematically analyze the RNA-Seq, ChIP-Seq, TMA, gene profiling and molecular signature of CRC cancer to get an insight of the main regulators and target genes in cancer. Our study first identified the DEGs between tumor and adjacent normal tissue mRNA expression in a large clinical patient cohort. These genes are filtered by the distance of TSS of ATF3 binding sites. Furthermore, our results suggested that the expressions of these target genes are more likely to be influenced by the ATF3 protein rather than mRNA (Figure 5). There was no significant relevance between ATF3 mRNA expression from database and OS. Here we focused on ATF3 protein expression in tumor cells, The TCGA mRNA expression came from mixed cell populations, which contributed to the ambiguity of expression due to subtype composition, especially for lower transcription factor expression. Next, we selected the transcription factor ATF3 and identified its targets using a filter method (Figure 1A). We showed that the pathway analysis result of CRC was consistent with known processes in CRC. In addition to this, we identified target genes that have not been previously reported and able to depict their possible roles. In summary, combining data from different sources is a promising strategy to identify the main regulators and target genes of cancer.

Notably, the result of the KEGG pathway analysis suggested that the DEGs with a ATF3 binding motif were functionally enriched on a developmental process, and a correlation existed between development and carcinogenesis. Expression of these target genes had a strong association with the OS in the CRC patients which was confirmed by Kaplan–Meier survival and Cox regression analyses (Figure 6 and Table 2, 3).

In the current study, with the expanded gene signatures, JASPAR database prediction identified ATF3 as one of the top overrepresented transcription factors, along with another key transcription factor STAT3 (Figure 2A). STAT3 is important and necessary for proliferation and survival in CRC-initiating cells [19]. Furthermore, these 11 gene signatures were validated in high ATF3-expressing cell lines (HCT116 and RKO) by targeting ATF3 with CRISPR editing. We found that 5 genes, including *CEACAM1, DUSP14, HDC, HLF* and *ULBP2*, were either upregulated or downregulated by ATF3 (Figure 3). Although 6 other genes, such as *TOMM34, STC2, PARM1, SPTBN2*, etc., had ATF3 binding motifs in their promoters, they were not regulated by ATF3 at least in these cell lines. *TOMM34* is reportedly involved in the growth of cancer cells and may contribute to the development of novel anticancer drugs and diagnosis of CRCs based on the immunohistochemistry results of *TOMM34* proteins in CRC tissues, which is more likely to be upregulated by amplification in CRC [20] rather than by transcriptional regulation through ATF3. Our data showed that the higher expression of ATF3 suppressed the carcinoembryonic antigen-related cell adhesion molecule 1 (*CEACAM1*) and hepatic leukemia factor (*HLF*). In addition, upregulated *HDC* and dual-specificity phosphatase 14 (*DUSP14*) are molecules that are related to cell adhesions with a low expression in the early stage of CRC, thus they act as tumor suppressors. However, *CEACAM1* is elevated in metastatic CRC, suggesting a role change in CRC progression [21]. However, our results are consistent with our model systems in which ATF3-regulated *CEACAM1* expression impacts metastatic CRC cells and survival outcome. From experimentation, histamine can conduct proliferation and angiogenesis in these cancer cells. Our KEGG pathway analysis of the ATF3-regulated target genes indicated that histidine metabolism is important for oncogenesis. After deleting ATF3 in the RKO cells, *HDC* expression *in vitro* was elevated and suggested a negative correlation between ATF3 and *HDC* in our study. The absence or down regulation of *HDC* by inhibition of mature myeloid cells, led to a higher rate of colon carcinogenesis, and as a result of this, CRC patients have a lower amount of histamine catabolism in their colonic mucosae [22]. The growth of tumor allografts was promoted by *HDC* deficient immature myeloid cells in the mice model and immature myeloid cells are differentiated after getting exogenous histamine. Thus, they have the ability to suppress growing tumor allografts [23]. All the evidences were from the *in vivo* experiments. ATF3 regulating *HDC* expressions in myeloid cells that contributed to the tumor growths may need to be validated for *in vivo* experiments in further studies. *DUSP14* is an important negative regulator of the mitogen-activated protein kinase (MAPK) signaling pathways, which are involved in inflammatory response, cancers, cell proliferation and differentiation [24]. Deleting ATF3 in RKO and HTC116 cells reduced the expression of *DUSP14* and influenced the proliferation rate (Figure 4A). *HLF* is an output regulator of circadian rhythms and is aberrantly expressed in human cancers [25], and our results suggested that it might be related to a cell death program. The ULBP2 gene is a known ligand of the activating NK receptor NKG2D, and it is critically involved in tumor immunosurveillance. In the RKO cell line, *ULBP2* was positively regulated by ATF3. On the other hand, *ULBP2* showed negative regulation, which indicates that ATF3 both positively and negatively regulates its target genes. This effect is also dependent on its binding with other proteins. Recently, we identified that ATF3 interacted with HDACs for epigenetic regulation (unpublished data), which also showed positive and negative regulation depending on the different HDAC isoforms.

The controversial roles of ATF3 were demonstrated to occur in both oncogenesis and tumor suppression. The most striking studies supported an oncogenic role of ATF3 based on correlative evidence of ATF3 overexpression in human cancer tissue [26]. The ATF3 expression is elevated in many human cancers, including breast, prostate, and Hodgkin lymphoma cancers. ATF3 may be oncogenic since it can protect cells from apoptosis and increase cell proliferation. Meanwhile, ATF3 can also promote metastasis of cell lines *in vitro* and *in vivo* by either overexpression or anti-sense/small interfering RNA knockdown [9]. When the ATF3 expression is expressed at lower levels in some cancers, such as esophageal squamous cell carcinoma and gastric cancer, ATF3 may act as a tumor suppressor [27]. ATF3 can also be induced through many anti-tumorigenic compounds, including a recently reported HDAC inhibitor [28] and non-steroidal anti-inflammatory drugs [29]. If ATF3 does act as a tumor suppressor, it may partially be due to the action of these compounds. However, after treating these compounds, the induction of ATF3 may result in deleterious effects if ATF3 acts as an oncogene [26]. ATF3 plays controversial roles both in oncogenesis and tumor suppression, which may be cell context dependent. In CRC models, ATF3 is a tumor suppressor through up-regulated heat-shock protein 90 (Hsp90) inhibition tumor growth [30]. However, two independent studies showed that ATF3 appears to promote tumor growth and migration in the experimental HT29 colon cancer model by either ATF3 antisense oligonucleotide or adenovirus-mediated overexpression of ATF3[16, 17]. Previous studies investigated a limited number of cell lines and small cohorts that were not biologically relevant. Thus, to fully characterize the function of ATF3 in tumor progression in CRC, we used an integrative strategy from ChIP-Seq, a larger database of gene profiling, molecular signatures with OS outcome and a TMA. The TMA was assessed with a specific antibody for ATF3 that was tested in colon tissues from our wild type and ATF3 null mice. Compared to the adjacent normal tissues, ATF3 was different due to its elevation in the CRC specimens. ATF3 overexpression is associated with a regulatory molecular signature, and this signature is functionally related to the pathogenic development of CRC from TCGA. Eleven of the genes with a molecular signature had putative ATF3 regulatory elements within their promoter by ChIP-Seq. Within these target genes, we demonstrated that ATF3 regulated genes, such as *CEACAM1, DUSP14, HDC, HLF* and *ULBP2* are the novel target genes for ATF3, after conducting our CRISPR-Cas9 genome editing experiment. Subsequently, some target genes impacted the invasion and proliferation of tumor cells *in vitro*. However, some genes need to be further investigated in a tumor environment using ATF3 knockout mice in CRC models to characterize the impact, such as *HDC* [22, 23].

Taken together, our results suggest that ATF3 promotes the invasion and proliferation of CRC cells, at least in part, via the regulation of CEACAM1-mediated EMT. *DUSP14*, *HDC*, and *HLF* mediated cell metabolism and proliferation. These findings advocate that ATF3, coupled with its target genes, is a prognostic marker for CRC progression.

## MATERIALS AND METHODS

### Patient clinical data and RNA-Seq data

All the clinical information and the RNA-Seq data were downloaded from the TCGA data portal (https://gdc-portal.nci.nih.gov/search/s?facetTab=cases). A total of 521 CRC samples (41 normal and 480 tumor) were analyzed. Two tumor samples were discarded in the subsequent analysis due to their incomplete clinical information. The RNA-Seq data for each sample was merged into a matrix containing counts of each Ensembl ID. The clinical data included gender, age, tumor stage, survival status, survival time and other demographic information (race, ethnicity, etc.).

### DEG analysis

Before the DEG analysis, the RNA-Seq count matrix had 60,483 Ensembl IDs. We used the GTF file (Homo_sapiens.GRCh38.86.chr) to obtain the gene symbol for each Ensembl ID, and 57,288 genes were kept for further study. The samples were separated into 4 groups by male/female and tumor/normal. We used the limma package [31] (version 3.26.9) in RStudio (version 3.2.0) with the voom method, linear modeling and empirical Bayes moderation to assess the DEGs. The genes expressed in at least 40 samples (the number of the smaller group) with count per million (CPM) >1 were kept for the downstream analysis. We used a cut-off of log-fold-change (log2FC) >1 and a False Discovery Rate (FDR) <0.05 to determine the final DEGs. The KEGG pathway and GO analysis were conducted in RStudio (version 3.2.0).

### Survival analysis for the DEGs

We used a Kaplan–Meier method on all the DEGs, using the median of the log-CPM to determine the high- and low-expression subgroups. We evaluated the significance of each subgroup by a log-rank test. The statistical analysis was performed with the RStudio survival package (version 2.40-1), and the P-value cut-off was set to 0.05.

### ATF3 binding site predicting

The JASPAR database was used to predict the potential ATF3 binding sites. The JASPAR CORE database contains a curated, non-redundant set of profiles that are derived from published collections of experimentally defined transcription factor binding sites for eukaryotes.

### ChIP-Seq data and peak annotation

The data from the ATF3 ChIP-Seq were downloaded from the GEO database (GSM1917774_1152) for the control group to identify the experimental ATF3 binding site. The ATF3-WT HCT116 colon cancer cell line was used for the ChIP analysis. The chromatin was sheared to an average fragment size of 500 bp by sonication using a Bioruptor and was immunoprecipitated using the indicated antibody. The sample was purified and sequenced using Illumina HiScanSQ following the protocols recommended by the manufacturer. The libraries were prepared according to Illumina's instructions. The reads were aligned to the hg19 reference human genome using bwa v0.6.1, and peak calling was performed by HOMER v4.1 using the GRCh37 (hg19) human genome. Finally, we selected genes having a peak within a certain range from the TSS using the IGV [32].

### TMA construction and cell lines

TMA of CRC tissues and adjacent tissues (HCol-Ade180Sur-03 for CRC) from 90 patients was purchased from Shanghai Outdo Biotech (Shanghai, China) [33]. All of the protocols using human specimens were approved by the Shanghai Jiao Tong University, and informed consent was obtained from all of the subjects. The TMAs contained all the patients’ clinicopathological information, including the patients’ gender, age, tumor site and size, clinical stage, TNM stage and follow-up date (ended in December 2011). Nine patients were excluded due to the inadequate completion of follow-up data and tissues. In total, 81 patients were included, with 41 males and 40 females of a median age of 70 (ranging from 24 to 90). The OS time ranged from 3 to 96 months. The TMAs were prepared and processed for immunostaining using an ATF3 (Santa Cruz, USA) antibody. The human colon cell lines SW480, SW48, DLD-1, HCT116, RKO and HT29 were purchased from ATCC (Manassas, VA). All of the cell lines were grown in RPMI-1640 medium with 10% fetal bovine serum (FBS) and 1% penicillin/streptomycin at 37°C with 5% CO_2_ in a cell culture incubator.

### Immunohistochemistry

Five-micrometer-thick TMA sections were deparaffinized in xylene and hydrated in a series of graded ethanol. Antigen retrieval was completed after heating in citrate buffer (pH 6.0) in a pressure cooker for 15 min. Endogenous peroxidases were blocked by 3% H_2_O_2_ for 10 min, and the sections were then incubated with goat serum for 20 min at room temperature. The slides were incubated with the ATF3 primary antibody (1:500 dilution in 1% bovine serum albumin PBS solution) at 4°C overnight and was then incubated with horseradish peroxidase (HRP) (Vectastain ABC kit, USA) for 30 min at room temperature. Finally, the slides were incubated with the ABC reagent for another 30 min at room temperature for signal amplification, washed with TBST and stained using 3, 3’-diaminobenzidine (DAB).

The staining results were scored by two pathologists blinded to the clinical data, and the staining was classified into four grades based on the staining intensity as follows: 0, no cell staining; 1, weak staining; 2, moderate staining; and 3, strong staining. The proportion of positively stained cells was classified into four levels as follows: level 1 (<25%); level 2 (25%-50%); level 3 (50%-75%); and level 4 (>75%). The final immunohistochemistry score was obtained by multiplying the intensity and proportion values (range 0-12), and the samples were grouped as 0 (score 0), 1 (score 1-3), 2 (score 3-6), 3 (score 6-9) and 4 (score 9-12) staining. For statistical purposes, final scores of 3 and 4 were defined as high expression and other scores as low expression.

### CRISPR

The MIT CRISPR design software was used to design the sgRNAs (http://crispr.mit.edu). The sgRNA ATF3 sequence was TCCCACTGTCAGCGACAGACCCCT. The lentiviral particles were produced by transient transfection of 293T cells grown in 10-cm Petri dishes with 10 μg of vector DNA containing a pFgh1tUTG vector with the target sgRNA inserted and the pFUCas9mCherry vector, which was gift from Marco Herold [34], along with the packaging constructs pMDL, pRSV-rev, and pVSV-G using standard calcium phosphate precipitation. The virus-containing supernatants were collected 48–72 h after transfection. To establish the ATF3 knockdown, HCT116 and RKO cells were treated with 8 ng/ml polybrene in the viral supernatant, and doxycycline hyclate (Sigma-Aldrich) was used to treat the cell lines to induce the expression of ATF3 sgRNA for 3 days. Cells without doxycycline hyclate treatment were used as the sgRNA control.

### Proliferation assay

The effect of ATF3 CRISPR on cellular growth and proliferation was determined *in vitro* using the PrestoBlue kit (Invitrogen, USA). Two thousand cells were seeded into 96-well plates with RPMI-1640 medium in a volume of 90 μl. After incubation, 10 μl of PrestoBlue reagent was added to each well, and after 30 min, the absorbance was read at a wavelength of 570 nm using a microplate spectrophotometer (BMG Labtech, Germany).

### Wound healing and invasion assays

Cell migration activity was assessed by a wound healing assay. Briefly, 10^5^ cells were seeded into 24-well plates and were incubated until they reached 90% confluency. The cells were starved for 24 h and were wounded by scratching lines with a 200-μl pipette tip. Cells were washed with PBS twice to remove the debris, then the cells were observed and photographed under a microscope. The images were recorded after 24 h and 48 h. A cell invasion assay was performed using Transwell chambers (24 well, 8 μm pore size, Corning, NY). In the assay, 600 μl of RPMI-1640 medium containing 20% FBS was used as the attractant in the lower chamber, and 100 μl of RPMI-1640 medium containing 5 × 10^5^ cells was added to the upper chamber; the cells were allowed to migrate through the pores. The cells on the lower side of the insert filter were fixed with 4% paraformaldehyde for 10 min and were stained with 0.1% crystal violet for 15 min. The numbers of cells on the underside of the filter from three randomly selected microscopic views were counted and photographed.

### Western blotting

Western blotting was performed using primary antibodies against ATF3 (1:500 dilution, Santa Cruz, USA) and actin (1: 10,000 dilution, Sigma, USA). The secondary antibodies were goat anti-rabbit IgG (1: 5,000 dilution, Invitrogen, USA) and goat anti-mouse IgG (1: 5,000 dilution, Invitrogen, USA) [35].

### Quantitative real-time PCR analysis

The quantitative real-time PCR analysis of the expression of *SPTBN2, DUSP14, CCNJL, CEACAM1, HLF, STC2, ULBP2, HDC, TOMM34, PARM1* and *C2orf48* was performed in triplicate on an Applied Biosystems 7700 Prism real-time PCR machine with SYBR Green QPCR mix and each gene probe. The expression of the target genes was normalized to the expression of the housekeeping gene 18S. To calculate fold change, the data were transformed using the ‘relative standard curve method and comparative threshold cycle (Ct) method (Δ ΔCt)’ as described by Applied Biosystems.

### Statistical analysis

The statistical analysis was performed using SPSS version 16.0 for Windows. A Kaplan–Meier plot and log-rank tests were used for the survival analysis. Univariate and multivariate Cox regression models were used to analyze the independent prognostic factors and the ATF3 candidate genes. The associations between ATF3 expression and the clinicopathological features were analyzed by Pearson's chi-square (χ^2^) test. The differences were considered significant at a value of p <0.05 [36, 37].

## SUPPLEMENTARY MATERIALS FIGURES AND TABLES




