# Supplementary material for: Overexpression of the transcription factor ATF3 with a regulatory molecular signature associates with the pathogenic development of colorectal cancer
Source: Oncotarget. 2017 Mar 29;8(29):47020–36. doi: 10.18632/oncotarget.16638 (PMC5564541; doi:10.18632/oncotarget.16638)
Supplement: Supplementary file 1 [file oncotarget-08-47020-s001.pdf]

Overexpression of the transcription factor ATF3 with a regulatory molecular signature associates with the pathogenic development of colorectal cancer

SUPPLEMENTARY MATERIALS

Quantitative RT-PCR

Quantitative real-time PCR (qRT-PCR) analysis was carried out to detect the mRNA expression. Total RNA extraction from colon cancer cells was performed with Trizol Reagent (Invitrogen). Then, 2 μg of total RNA was reverse-transcribed with high-capacity cDNA reverse transcription kits (Applied Biosystems, USA). The primers below were used to amplify ATF3, SPTBN2, DUSP14, CCNJL, CEACAM1, HLF, STC2, ULBP2, HDC, TOMM34, PARM1 and C2orf48. RT-PCR primers:

ATF3:	F: 5’- GAGGATTTTGCTAACCTGACACC-3’
	R: 5’- TTGACGGTAACTGACTCCAGC-3’
SPTBN2:	F: 5’-GAGGTCTCGCATTAAGGCTCT-3’
	R: 5’-CTTTGGCAGTATCTCTCCCGA-3’
DUSP14:	F: 5’-TCAACTGGCCCCAATTTGAGT-3’
	R: 5’-CATCAGGTACGCGATACACAG-3’
CCNJL:	F: 5’-CCTGCGCGAGAAGGAACTG-3’
	R: 5’-CGTTGTAGCGATCCATGAAGTG-3’
CEACAM1:	F: 5’-TGCTCTGATAGCAGTAGCCCT-3’
	R: 5’-TGCCGGTCTTCCCGAAATG-3’
HLF:	F: 5’-CCACCTTTATCCCGCCTCC-3’
	R: 5’-TTTACTAAATGCGTCTTCGTGGT-3’
STC2:	F: 5’-ACAGGTTCGGCTGCATAAGC-3’
	R: 5’-GAGGTCCACGTAGGGTTCG-3’
ULBP2:	F: 5’-AGCAACTGCGTGACATTCAG-3’
	R: 5’-GCCATCCTATACAGTCTCCCA-3’
HDC:	F: 5’-ATGCACGCCTACTACCCAG-3’
	R: 5’-CAGTCCATGACGTTCATCTCC-3’
TOMM34:	F: 5’-AAGCCAGAGTTCTGAAGGAAGAAGG-3’
	R: 5’-AGCTTGAGGGCTTCTGTGCAGT-3’
PARM1:	F: 5’-GTACAGAGTCTGCCTACATCAGC-3’
	R: 5’-TGGAGAGCTAGTCCAGATGGT-3’
C2orf48:	F: 5’-CAGTGTGCGGGGATGAGAAG-3’
	R: 5’-GGGGATTCCCTTTCAAGACGTA-3’

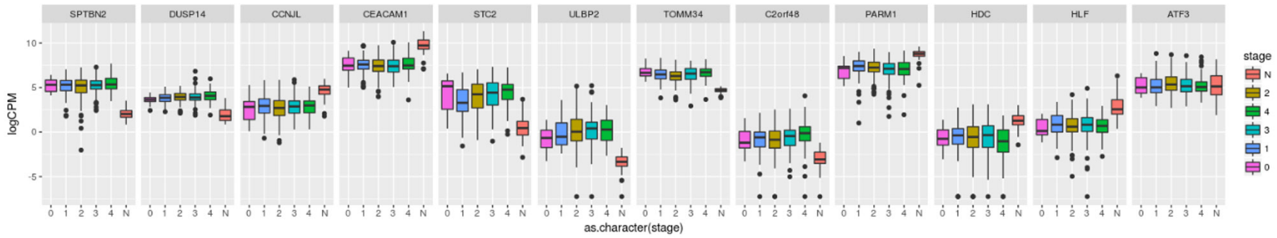

Supplementary Figure 1: ATF3 target genes expression across different clinical stages. Y-axis is the logCPM, x-axis is clinical stages (indicated by different colors)

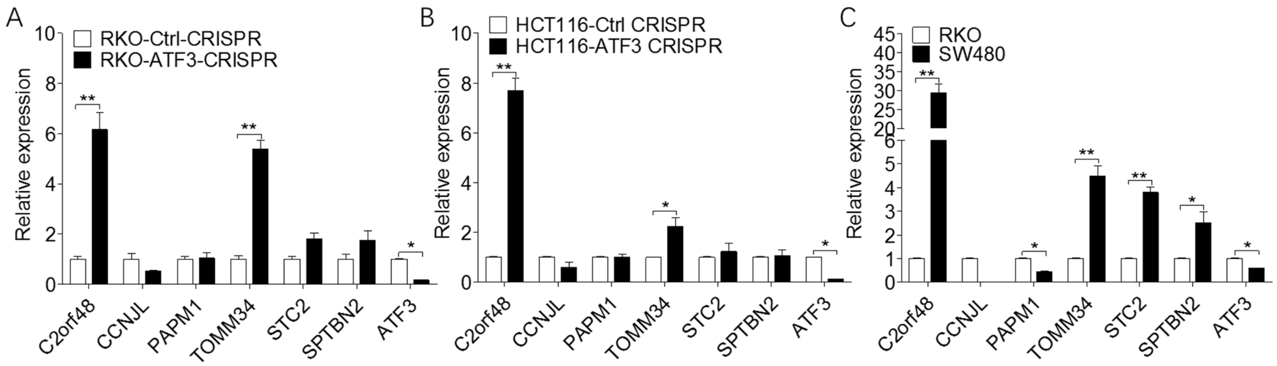

Supplementary Figure 2: mRNA expression of different genes by qRT-PCR analysis.

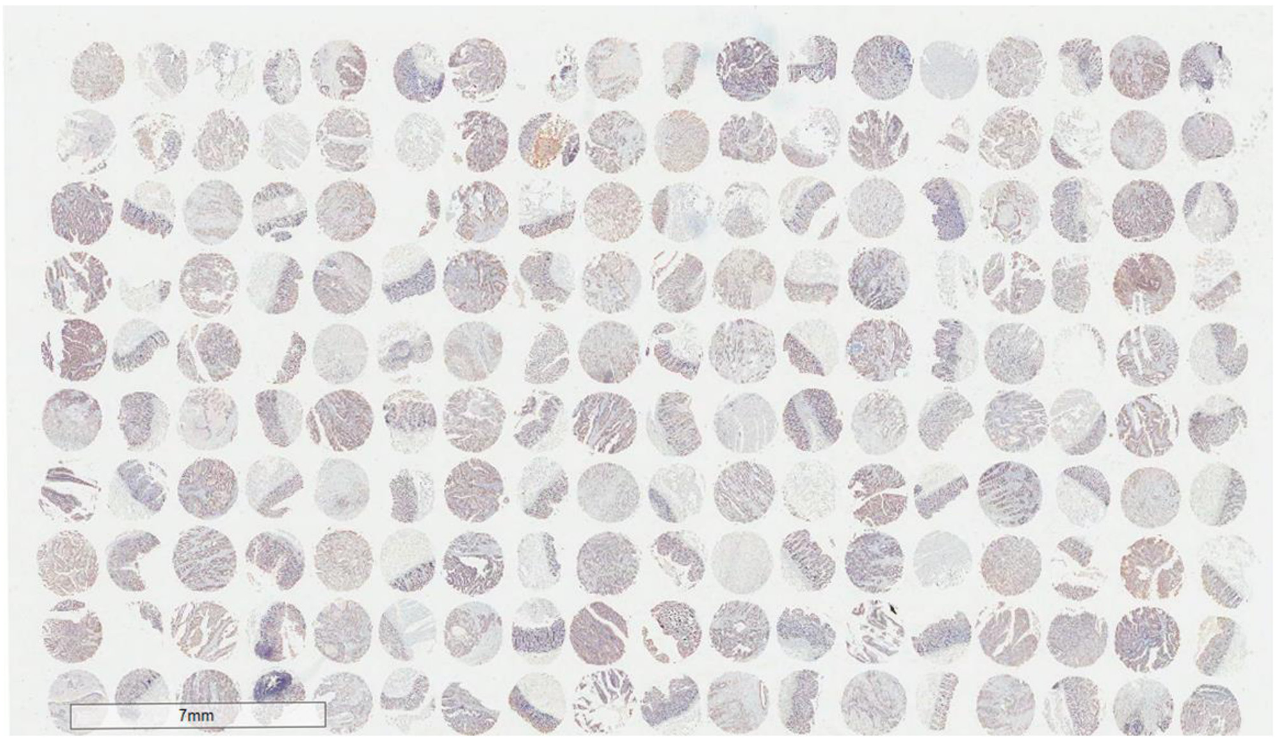

Supplementary Figure 3: Scanning image of the whole slide by Aperio ScanScope slide scanner.

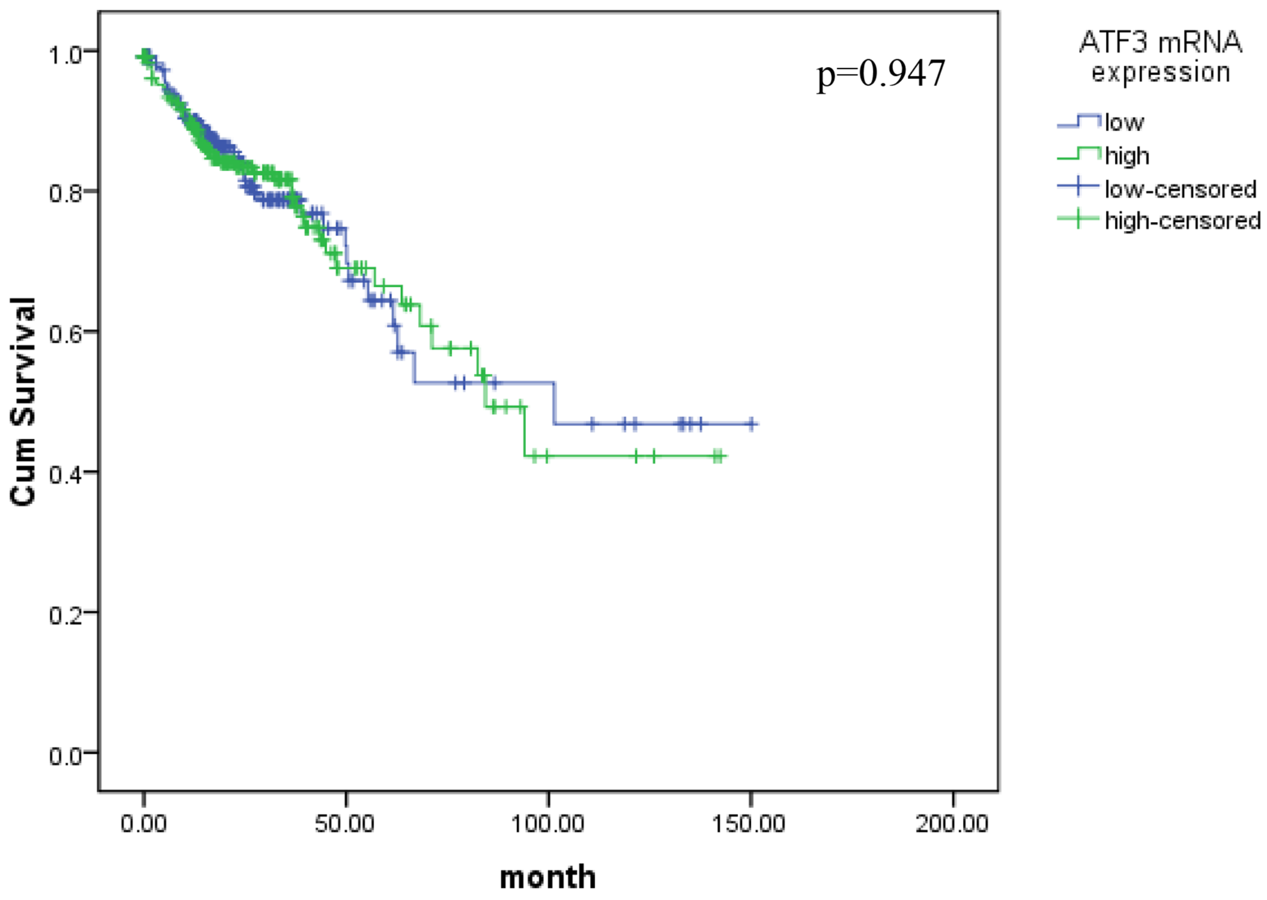

Supplementary Figure 4: Kaplan Meier curve for ATF3 mRNA expression from TCGA database.

Supplementary Table 1: Complete information of 47 genes from TCGA and ChIP-Seq data

See Supplementary File 1

Supplementary Table 2: ATF3 expression in CRC tumor tissues and adjacent normal tissues

	CRC tumor tissues	Adjacent normal tissues	Pearson χ^2	p value
ATF3$^+$	23	0		
ATF3$^-$	58	81		
Positive rate (%)	23 (28.3%)	0 (0%)	26.806	<0.001*

χ^2 test for between-group analysis.
* $p < 0.05$ indicates statistical significance.
