# Supplementary material for: Overexpression of the transcription factor ATF3 with a regulatory molecular signature associates with the pathogenic development of colorectal cancer
Source: Oncotarget. 2017 Mar 29;8(29):47020–36. doi: 10.18632/oncotarget.16638 (PMC5564541; doi:10.18632/oncotarget.16638)
Supplement: Supplementary file 2 [file oncotarget-08-47020-s002.pdf]

Overexpression of the transcription factor ATF3 with a regulatory molecular signature associates with the pathogenic development of colorectal cancer

Supplementary Table 1. Complete information of 47 genes from TCGA and ChIP-Seq data

Gene Symbol	Entrezid	Chr	Male				Female				Distance.to.TSS	p.value-survival	comments
			logFC	AveExpr	P.Value	adj.P.Val	logFC	AveExpr	P.Value	adj.P.Val			
NPY6R	4888	chr5	-5.353874	-2.302335	1.53E-34	2.20E-32	-5.7417409	-2.3023349	5.20E-46	1.40E-43	32438	0.002	
PCOLCE2	26577	chr3	-4.405367	-1.032092	1.60E-26	1.45E-24	-4.5505777	-1.0320918	2.83E-29	3.07E-27	-7185	0.002	
NLGN1	22871	chr3	-4.296365	-3.285662	4.92E-31	5.90E-29	-3.8350618	-3.2856622	3.72E-23	2.87E-21	-61991	0.023	
WDR78	79819	chr1	-2.232263	1.4526828	2.25E-11	6.18E-10	-2.5910948	1.45268283	3.67E-19	2.07E-17	57226	0.027	INTRON
B3GNT6	192134	chr11	-4.426439	1.4926097	9.58E-13	3.08E-11	-4.9030286	1.49260974	3.96E-16	1.67E-14	-18508	0.012	
SPTBN2	6712	chr11	2.9693281	4.8952155	2.58E-10	6.27E-09	2.96550493	4.89521555	1.21E-10	2.97E-09	1031	0.045	
DUSP14	11072	chr17	1.4875704	3.764131	0.001909	0.01688	2.16291873	3.76413099	1.46E-10	3.52E-09	-888	0.042	
CCNJL	79616	chr5	-1.57743	2.9131861	0.003564	0.02944	-2.2672314	2.91318605	3.91E-10	8.99E-09	-3561	0.046	
CEACAM1	634	chr19	-2.198474	7.6232836	2.28E-06	3.45E-05	-2.578757	7.62328357	7.72E-10	1.74E-08	-2968	0.019	
VAT1L	57687	chr16	-2.579783	0.8015917	3.13E-15	1.28E-13	-2.229431	0.80159172	8.66E-10	1.94E-08	89389	0.042	INTRON
ZBTB7C	201501	chr18	-2.863384	3.739254	6.63E-08	1.25E-06	-3.0163186	3.739254	3.89E-09	8.11E-08	-64144	0.019	
NAT2	10	chr8	-2.126481	1.8604587	2.57E-05	0.00033	-2.4803398	1.86045865	8.48E-09	1.69E-07	35258	0.036	
ARNTL2	56938	chr12	2.1845062	4.4464967	1.88E-06	2.89E-05	2.45242276	4.44649668	1.25E-08	2.46E-07	285	0.043	
SIGLEC1	6614	chr20	-2.214853	2.4120399	3.70E-06	5.43E-05	-2.4034977	2.41203987	2.17E-08	4.14E-07	-9192	0.047	
ELFN1-AS1	101927125	NA	4.4322997	2.0992975	1.21E-07	2.22E-06	4.53104358	2.09929747	2.80E-08	5.27E-07	319	0.009	NO MAP
EIF4E3	317649	chr3	-1.931843	4.313934	8.14E-07	1.32E-05	-2.0144012	4.313934	5.83E-08	1.05E-06	651	0.034	
CNN1	1264	chr19	-4.070283	4.4898023	1.12E-13	4.00E-12	-3.072749	4.48980229	9.02E-08	1.58E-06	-2533	0.007	
TLR10	81793	chr4	-2.646391	-0.981597	6.92E-09	1.45E-07	-2.4876992	-0.9815971	1.12E-07	1.95E-06	24823	0.036	
SPEG	10290	chr2	-3.124376	1.7152555	5.04E-14	1.85E-12	-2.4554688	1.7152555	2.44E-07	4.00E-06	-7727	0.009	
HLF	3131	chr17	-2.281261	0.8936062	1.11E-09	2.53E-08	-2.0433738	0.89360622	9.45E-07	1.42E-05	-102608	0.016	
STC2	8614	chr5	3.2839862	3.7588117	2.47E-05	0.00032	3.76721921	3.75881175	1.15E-06	1.70E-05	-351	0.047	
ULBP2	80328	chr6	3.4528277	-0.049079	8.58E-06	0.00012	3.52613126	-0.0490794	1.98E-06	2.82E-05	-7493	0.005	
PCDH9	5101	chr13	-2.625792	-1.266862	4.23E-08	8.10E-07	-2.3995051	-1.2668625	2.44E-06	3.42E-05	110642	0.03	
ATOH1	474	chr4	-2.891656	2.7942974	1.14E-05	0.00015	-2.9467636	2.79429741	4.09E-06	5.56E-05	-650467	0.008	
ATP8B1	5205	chr18	-1.599663	7.4251985	0.00015	0.00167	-1.7318411	7.42519845	4.61E-06	6.22E-05	-17117	0.008	
SMOX	54498	chr20	2.1145197	4.2872456	8.10E-06	0.00011	2.1229295	4.28724561	6.19E-06	8.19E-05	-12301	0.008	
HDC	3067	chr15	-1.924886	-0.47529	0.000853	0.00813	-2.1443704	-0.4752898	3.53E-05	0.000419	7901	0.017	INTRON
STK31	56164	chr7	3.0791919	0.7862269	2.45E-05	0.00032	3.00922849	0.78622686	3.59E-05	0.000425	154886	0.048	
TNFAIP8L3	388121	chr15	-1.816902	1.3121712	4.52E-05	0.00056	-1.7822138	1.3121712	7.82E-05	0.000876	-16006	0.011	
FCRL5	83416	chr1	-2.654324	0.9017842	6.09E-06	8.67E-05	-2.4423437	0.90178418	9.47E-05	0.00104	56257	0.032	
KIF14	9928	chr1	1.8946204	3.2770271	6.70E-05	0.0008	1.85582922	3.27702713	9.82E-05	0.001075	-19414	0.049	
CXCL8	3576	chr4	4.1240086	5.2281824	1.12E-05	0.00015	3.45706307	5.22818238	0.000125	0.001338	-35759	0.002	
TOMM34	10953	chr20	1.6442229	6.3006993	0.000138	0.00156	1.5984586	6.30069927	0.000277	0.002768	-93	0.027	
FENDRR	400550	chr16	-2.39868	3.113129	3.69E-08	7.13E-07	-1.8894581	3.11312904	0.000287	0.002844	29177	0.03	INTRON
GLDN	342035	chr15	-2.156141	0.3656588	3.12E-06	4.65E-05	-1.8887583	0.36565884	0.000316	0.00312	108632	0.003	
RIMS3	9783	chr1	-1.764196	2.4025066	0.000376	0.00387	-1.7424555	2.40250665	0.000395	0.003824	19063	0.018	INTRON
CKAP2L	150468	chr2	1.5734772	3.7951892	0.000896	0.0085	1.58374538	3.79518922	0.00059	0.005513	27479	0.024	
MELK	9833	chr9	1.479857	4.782172	0.00214	0.01867	1.52658801	4.78217203	0.000651	0.006034	12617	0.043	
MPP2	4355	chr17	-2.270673	0.3716658	5.18E-11	1.39E-09	-1.6489736	0.37166577	0.000665	0.00615	1176	0	
C2orf48	348738	chr2	2.1623021	-0.839182	0.00122	0.01121	2.18648862	-0.8391819	0.000897	0.008071	-5185	0.02	
SFRP2	6423	chr4	-2.699162	4.1099248	0.001609	0.01446	-2.7379806	4.1099248	0.00112	0.009857	-41556	0.05	
SPTBN4	57731	chr19	-1.901826	-1.223642	0.000199	0.00216	-1.7349929	-1.2236416	0.001912	0.01603	-1092	0.01	

MAD2L1	4085	chr4	1.4956752	5.4226633	0.003826	0.03136	1.52019743	5.42266334	0.002205	0.018239	-262720	0.041	
SERTAD4	56256	chr1	-2.073672	1.5507117	1.03E-05	0.00014	-1.7193806	1.55071166	0.002305	0.018984	-44416	0.036	
DTNA	1837	chr18	-2.250072	1.2587594	1.01E-05	0.00014	-1.8616094	1.25875937	0.002423	0.019835	-2366	0.019	
MIR4435-2HG	541471	chr2	1.5928041	3.7391011	0.000329	0.00343	1.46872645	3.73910112	0.002465	0.020114	15	0.038	NO MAP
PARM1	25849	chr4	-1.682999	7.2208161	0.003938	0.03214	-1.698484	7.22081608	0.002919	0.023278	-198	0.031	
